# Comparative Seed Morphology of Tropical and Temperate Orchid Species with Different Growth Habits

**DOI:** 10.3390/plants9020161

**Published:** 2020-01-29

**Authors:** Surya Diantina, Craig McGill, James Millner, Jayanthi Nadarajan, Hugh W. Pritchard, Andrea Clavijo McCormick

**Affiliations:** 1 School of Agriculture and Environment, Massey University, Tennent Drive, 4410 Palmerston North, New Zealand; c.r.mcgill@massey.ac.nz (C.M.); j.p.millner@massey.ac.nz (J.M.); a.c.mccormick@massey.ac.nz (A.C.M.); 2 Indonesia Agency for Agricultural Research and Development (IAARD), Jl. Ragunan 29, Pasar Minggu, Jakarta Selatan 12540, Indonesia; 3 The New Zealand Institute for Plant and Food Research Limited, Batchelar Road, Fitzherbert, 4474 Palmerston North, New Zealand; jayanthi.nadarajan@plantandfood.co.nz; 4 Royal Botanic Gardens Kew, Wellcome Trust Millennium Building, Wakehurst, Ardingly, West Sussex RH17 6TN, UK; h.pritchard@kew.org

**Keywords:** air-space, epiphytic, terrestrial, tropical, temperate, micro-morphometric

## Abstract

Seed morphology underpins many critical biological and ecological processes, such as seed dormancy and germination, dispersal, and persistence. It is also a valuable taxonomic trait that can provide information about plant evolution and adaptations to different ecological niches. This study characterised and compared various seed morphological traits, i.e., seed and pod shape, seed colour and size, embryo size, and air volume for six orchid species; and explored whether taxonomy, biogeographical origin, or growth habit are important determinants of seed morphology. We investigated this on two tropical epiphytic orchid species from Indonesia (*Dendrobium strebloceras* and *D. lineale*), and four temperate species from New Zealand, terrestrial *Gastrodia cunnninghamii*, *Pterostylis banksii* and *Thelymitra nervosa*, and epiphytic *D. cunninghamii*. Our results show some similarities among related species in their pod shape and colour, and seed colouration. All the species studied have scobiform or fusiform seeds and prolate-spheroid embryos. Specifically, *D. strebloceras*, *G. cunninghamii*, and *P. banksii* have an elongated seed shape, while *T. nervosa* has truncated seeds. Interestingly, we observed high variability in the micro-morphological seed characteristics of these orchid species, unrelated to their taxonomy, biogeographical origin, or growth habit, suggesting different ecological adaptations possibly reflecting their modes of dispersal.

## 1. Introduction

Most orchid species are endangered because of over collection and loss of habitat, so there is an urgent need to develop techniques to conserve them both *in situ* as well as *ex situ* [1]. Seed morphology is an important trait related to biological and ecological processes such as seed dormancy and germination [2,3], adaptation to habitat for seed dispersal [4,5,6,7], and seed storability [8]. Therefore, investigating seed morphology is relevant to understanding the plant’s reproduction under natural conditions and to devise optimal protocols for seed storage and propagation.

Seed morphology can also provide relevant information about orchid evolution and adaptations. This information could be particularly useful in comparative studies, since seed characters are considered inherent traits, being more conservative than other features. Previous studies have used seed morphology to investigate taxonomic, phylogenetic, and phytogeographic relationships among orchid species [6,9,10,11,12,13,14].

Orchid seeds are extremely light and small, compared with those in other plant families, and are produced in large numbers (reaching up to a million) inside a seed pod [6]. A unique characteristic of orchid seeds is that instead of an endosperm, there is an ‘air-space’ surrounding a small globular embryo within a membranous testa. The air-space volume in orchid seeds varies depending on the species. There is a relationship between seed size and embryo volume that determines the proportion of air-space [6,9]. Moreover, Arditti [6] and Leck [15] suggested that the amount of air trapped influences seed dispersal by affecting floatability (in air and water) and buoyancy, reflecting ecological adaptations to different distribution ranges (which is relevant to *in situ* conservation). For this reason, studies investigating the air-space within the seed and its relation to taxonomy and living habitat are important to identify the best conservation strategy.

Historically, orchid species have been classified into five subfamilies by floral morphological characters [16]. Among those five subfamilies, Epidendroideae and Orchidoideae are the largest in the orchid family (Orchidaceae), comprising 84% and 14% of the described orchids, respectively [11]. Most orchids belonging to the subfamily Epidendroideae are tropical epiphytic orchids [17]. In contrast, most of the Orchidoideae occupy an ecological niche as temperate terrestrial species with mycorrhizal symbionts [11]. Therefore, it is possible that species belonging to these two subfamilies have different adaptations to their ecological niches.

Indonesia, a tropical country, is the habitat of a great diversity of orchids, with roughly 5000 species [18], most of which are epiphytic. In contrast, New Zealand is temperate, having around 120 orchid species in 30 genera. These are predominantly terrestrial, and over half are endemic [19,20].

This study characterised and compared the morphological seed traits of two tropical epiphytic species, *Dendrobium strebloceras* and *D*. *lineale* from Indonesia (subfamily Epidendroideae) and four temperate species from New Zealand, the endemic epiphytic orchid *D*. *cunninghamii* (Epidendroideae) and terrestrial orchids *Gastrodia cunnninghamii* (Epidendroideae), *Pterostylis banksii* (Orchidoideae), *Thelymitra nervosa* (Orchidoideae). The aim of the study was to determine whether taxonomy, biogeographical origin, or growth habit of the species are important determinants of orchid seed morphology.

## 2. Results

### 2.1. Pod and Seed Morphology

**Figure 1:**
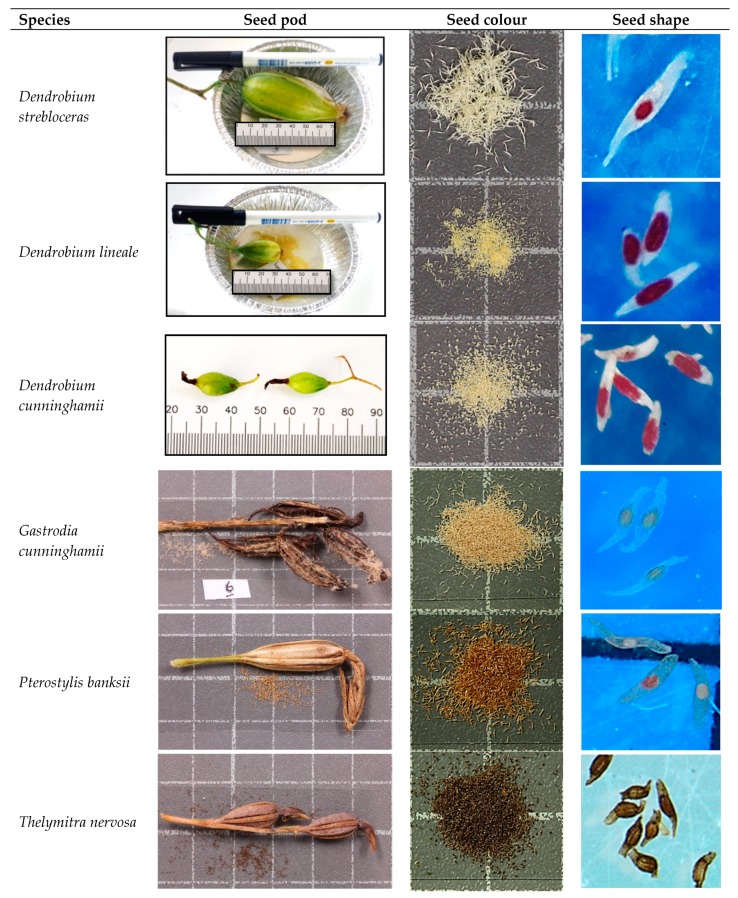
Morphological characteristics of orchid pods and seeds. The first column indicates the orchid species, the second column shows the colour and approximate size of seed pods with white squares representing one centimetre. The third column shows the approximate size and colour of the seeds (white square = 1 cm) and the fourth column shows a light microscope image of the seeds.

*Dendrobium* species produced yellow-green seed pods when mature (Figure 1), while the other three genera; *Gastrodia*, *Pterostylis*, *Thelymitra*, produced brown pods with small differences in hue at maturity. In the subfamily of Epidendroideae, a large variation in pod length was found in the tribe *Dendrobieae* (Table 1). *D. strebloceras* produced the largest seed pods, followed by *D. lineale*, while *D. cunninghamii* was found to produce the smallest pods. Terrestrial temperate species, *G. cunninghamii* (Epidendroideae), *P. banksii and T. nervosa* (Orchidoideae) produced intermediate-sized pods. All pods split after seeds inside the pod reached maturity. Seeds in the *Dendrobium* species were generally yellow with slight differences in intensity; the remaining genera produced brown seeds that also varied in tone intensity, from light to dark brown. Moreover, during seed morphology assessment, we found that seeds of *D. strebloceras* and *P. banksii* had a sticky or adhesive surface.

Irrespective of their taxonomy or ecological habitat, most species in this study had a fusiform or scobiform seed shape and prolate-spheroid embryos (Figure 1). Specifically, *D. strebloceras*, *G. cunninghamii*, and *P. banksii* had an elongated-balloon shape and *T. nervosa* had a truncated seed shape, tapered at one end and blunt at the other end. 

### 2.2. Seed Micromorphology Assessment

A general linear model (GLM) revealed no effect of taxonomy (Epidendroideae or Orchidoideae), geographical origin (tropical or temperate) or habit (epiphytic or terrestrial) on seed traits. However, all micro-morphological traits varied significantly among species (Pillai’s Trace F = 173.439, df = 566, *p* < 0.001). The micro-morphological data for the six species are shown in Table 2.

We also calculated the relationship (ratio) between some of the parameters investigated (Table 3), i.e., Embryo Length (EL) and Embryo Width (EW), Seed Length (SL) and Seed Width (SW), and Seed Volume (SV) and Embryo Volume (EV) to further explore the occurrence of morphological trends related to taxonomy, origin, and habit, with a similar outcome (i.e., only species has a significant effect F = 97.601, df = 882, *p* < 0.001).

Although we did not find specific traits related to orchid taxonomy, origin, or habit; most Epidendroideae observed in this study (*D. lineale*, *D. cunninghamii* and *G. cunninghamii*) had a high EL/EW ratio (>2), and low embryo volume. In contrast, the Orchidoideae had relatively low EL/EW ratio and high EV. *D. strebloceras* had the lowest EL/EW ratio and highest EV of all species. We did not find similar trends for other traits; however, we consistently observed that *D. strebloceras* had different morphological traits than the other species, even those in the same genus (Table 2 and Table 3).

Elongated seeds (SL/SW ratio > 5) were found in *G. cunninghamii*, *P. banksii* and *D. strebloceras*, respectively (Table 3). While *D. lineale*, *D. cunninghamii* and *T. nervosa* had truncated seeds (SL/SW ratio < 5). Following the classification in Barthlott et al. [9], *D. strebloceras* and *Pterostylis banksii* produced large seeds. *G. cunninghamii* had medium-sized, but elongated seeds, with the biggest SL/SW ratio. On the other hand, *T. nervosa* was also classified as a medium seed (Table 3).

*T. nervosa* had a bigger seed and embryo volume than *D. lineale* but a lower SV/EV ratio, explained by the high occupancy of the embryo, resulting in a low air volume within the seed. Seeds of *D. cunninghamii*, *G. cunnninghamii*, and *P. banksii* had intermediate SV/EV ratios with higher airspace percentages. *D. strebloceras* had the highest SV/EV ratio and the largest airspace of the six studied species (Table 2 and Table 3).

When comparing *Dendrobium* species (Table 2 and Table 3), *D. lineale*, a tropical epiphytic-lithophytic, had a similar embryo size as that of *D. cunninghamii*, a temperate epiphytic-lithophytic, but *D. lineale* had a significantly smaller air-space compared with *D. cunninghamii.* In contrast, *D. strebloceras*, which has a similar habitat as *D. lineale* had significantly higher values for most parameters measured than the other *Dendrobium* species.

*T. nervosa* and *G. cunninghamii* did not differ in their seed volume, but *T. nervosa* had a larger EV, and consequently, a smaller air-space than *G. cunninghamii*. On the other hand, although *P. banksii* has a smaller embryo size than *T. nervosa*, a comparison of seed volume showed that *P. banksii* had the biggest SV, which was 2.25-fold higher than either *T. nervosa* or *G. cunninghamii*, thus the largest airspace among the temperate species in this study (Table 2).

## 3. Discussion

We characterised and compared pod and seed morphological traits of six orchid species. Our results show that pod appearance and colour can be good taxonomic indicators, while there is high variability in seed measurements (especially at the micromorphological level), suggesting that these traits are species-specific, possibly reflecting different modes of seed dispersal, and might not be suitable to identify taxonomical relationships. However, micromorphological traits can be useful to prioritise species for conservation and select appropriate *in situ* and *ex situ* conservation strategies.

We found similarities within the genus *Dendrobium* in their pod qualitative morphology; in particular their pod appearance and colour. Many other *Dendrobium* species share similar traits [18], suggesting that these features are useful taxonomic criteria to identify *Dendrobium* species. Similarity in shape of testa and seed pigmentation (yellow colour) have also been reported in most *Dendrobium* species [21], thus may be important characters for taxonomic markers, reflecting their close phylogenetic relationship. However, Barthlott et al. [9] suggest that seed colour is not a reliable taxonomic trait, as inconsistencies can be found within clades or genera, except in *Diuris* clade (a terrestrial orchid genus restricted to Australia, which has a very characteristic seed appearance with dark brown colour).

This study provides evidence of significant variability between species at the micro-morphometric level, independently of their genus, distribution range, and habit. Differences in seed morphology (coating, shape, weight and air volume) may reflect variations in their dispersal mechanisms or adaptations to different environments [6,22,23]. For instance, *P. banksii* and *D. strebloceras* belong to different genera, and have different growth habits and distribution ranges; but share several seed traits that facilitate dispersion. Both have a large air volume, allowing longer seed floatation time in the air [6,22], elongated seeds that disperse further than truncated seeds [24] and sticky or adhesive surfaces can assist animal-mediated seed dispersal [25].

Observations of seed qualitative morphology showed that species in this study had a fusiform testa or prolate-spheroid embryo, which are the most common shapes in the Orchidaceae family. The elongated seed shape in *D. strebloceras*, *G. cunninghamii*, and *P. banksii*, is not predominant among orchids but has been reported for other orchid species of unrelated taxonomic groups, which suggests that it may be a result of different adaptations to their habitats [13].

Our findings support the results of Wang et al. [21], who suggest that seed morphometric has no relationship with division of section in *Dendrobium*. However, Lavarack et al. [17] proposed that *Dendrobium* species are characterised by having very small seeds (<5 mm long), with some exceptions in the *Spatulata* section (which includes *D. strebloceras*). Therefore, more research is needed to confirm an association between seed morphometry and taxonomy in *Dendrobium* and the other groups.

According to Arditti and Ghani [6], the relationship between orchid seed dimension and air-space percentage is closely connected with their ecological adaptations. Recent findings by Chaudhary et al. [26] suggest that *Dendrobium* species from temperate regions require a higher air-space than species from sub-tropical or tropical regions, to facilitate buoyancy for optimal seed dispersal (an ecological adaptation to low atmospheric pressure). We found that *D. cunninghamii* (temperate) had a bigger (two-fold) air-space percentage than *D. lineale* (tropical). Moreover, Prasongsom et al. [2] also reported the air-space percentage of nine tropical *Dendrobium* species from Thailand fell in a range of 12.8%–36.3%. However, seed morphometric characters of *D. strebloceras* and *D. lineale* were significantly different, albeit they shared the same ecological habitat and taxonomic traits; both being epiphytic, large plants, with large flower size, present in low-altitude tropical rain forests. It is possible that, *D. strebloceras* is an exception to the rule together with other members of the same clade [17]. Therefore, more research is required to understand the ecological or evolutionary conditions that led this species (and probably others in the *Spatula* section) to develop larger seed sizes and air volumes.

Despite its minuscule size, the embryo is a crucial element that determines airspace proportion within the seed, thus seed buoyancy, floatation time and dispersal. There is evidence that bigger embryo volume is positively correlated to seed weight [27], determining seed ability to float on air and distribution [6]. EL/EW ratios above 1 show that all orchid species in this study have prolate-spheroid shaped embryos. Orchids from the subfamily Epidendroideae had higher EL/EW ratios (except *D. strebloceras*) than those in the subfamily Orchidoideae. Observations of seed volume showed that the Epidendroideae group has smaller seed volumes than Orchidoideae (except *D. Strebloceras*), further supporting the hypothesis that *D. strebloceras* has atypical morphological traits [17].

According to Verma et al. [13], terrestrial species have bigger air-spaces because of their bigger seed volume, thus bigger SV/EV ratio than epiphytic species. Moreover, seeds with SV/EV ratios above 2.2 were suggested to be more buoyant than those with lower ratios, thus enabling wider plant distribution ranges. Our data support these findings for the temperate terrestrial species *D. cunninghamii*, *G. cunninghamii* and *P. banksii*, compared with the tropical epiphytic *D. lineale*. Nonetheless, the terrestrial orchid *T. nervosa* did not follow the same pattern. This species has a comparatively big embryo volume, thus low SV/EV ratio and low air volume. A similar pattern was found in *Paphiopedillum* sp., which has limited seed dispersal and shorter buoyancy periods than other terrestrial species, consequently having a restricted distribution area [6].

Plant adaptations to specific or limited geographic distribution ranges stimulate the evolution of a wide variety of morphological characters, including seed dispersal mechanisms [28]. Our study reflects such variability, indicating that even closely related species may have different seed morphologies associated to their optimal dispersal strategies. Based on the seed dispersal characteristics proposed by Howe [23], it is possible to speculate that *D. lineale*, being epiphytic and having small seeds with low air percentages, is better suited for water dispersal (hydrochory) along the coastal of New Guinea. The wax-coated testa in *Dendrobiinae* [4] may be an advantage for seed distribution along the coastal stream. In contrast, bigger *D. strebloceras* seeds with a large air-space and high SV/EV ratio may rely on wind dispersal or take advantage of their adhesive surface to better attach them to the bark of the trees or use animals as dispersers (zoochory); which is suggested to be the most effective seed dispersal below closed canopies [29].

Regarding terrestrial species, Lechnebach and Robertson [30] suggested wind-dispersal as the main vector in *G. cunninghamii* and related the ability of seeds to easily be carried by wind with the widespread distribution of this species in the North and South Islands of New Zealand, and on the Stewart and Chatham Islands [31]. In the same study, the authors investigated other terrestrial orchids from the genera *Pterostylis* and *Thelymitra;* and found that *Thelymitra* was self-pollinated, while *Pterostylis* was cross-pollinated and heavily reliant on insect vectors. This may explain the larger seed sizes and adhesive surfaces in *Pterostylis* and the low air volume in *Thelymitra*.

Overall, our results show similarities in seed pod colour and shape between orchid genera, but a high diversity at the micro morphological level, where ecological traits (e.g., seed dispersal) rather than taxonomy, biogeographical origin, or growth habit are likely to determine seed morphology. We strongly encourage further studies including more orchid species to validate these results.

## 4. Materials and Methods

### 4.1. Seed Materials

Seeds of six orchid species from Indonesia and New Zealand were collected from mature and naturally dehiscing capsules. Two orchid species from tropical Indonesia, an epiphytic-lithopytic *Dendrobium lineale* (morebe shower/kui blue) and epiphytic *Dendrobium strebloceras* (twist-horn *Dendrobium*), were obtained from the IAARD (Indonesia Agency for Agricultural Research and Development) collection. They were hand-pollinated and grown in glasshouse conditions at Cipanas Experimental Field, West Java (tropical rainforest climate, 19 ± 6 °C, ≈1100 m above sea level) and harvested at maturity, about 5 months after pollination. One epiphytic-lithophytic temperate orchid, *D. cunninghamii* (winika/pekapeka), was obtained from Pukeiti Forest, North Island, New Zealand (temperate rainforest climate, 8 ± 5 °C, ~490 masl).

Seed pods of three terrestrial New Zealand orchids, *Gastrodia cunninghamii* (potato orchid/huperei), *Pterostylis banksii* (greenhood orchid/tutukiwi) and *Thelymitra nervosa* (spotted sun orchid), were collected from Iwatahi Native Orchid Heritage Protection Area under a *Pinus nigra* plantation at Kaingaroa forest, Taupo Napier road, North Island (marine west coast climate, 11 ± 5 °C, ~544 masl). The plant taxonomy, morphology, biogeographical origin, and ecological habits for each species are described in Table S1.

### 4.2. Evaluation of Seed Morphological Variability

Seed pod shape and seed colour were visually assessed and recorded. They were classified based on the general orchid seed colour proposed by Barthlott et al. [9]. Seed pod length was measured with a ruler and pictures of the seeds were taken using a Nikon DSLR camera on a copy stand with 1 cm grid (Kaiser RS1, Germany). To improve visibility, seeds were stained with a reagent, tetrazolium chloride, following the procedure by Hosomi et al. [32]. Both seed and embryo shapes and measurements were taken using a light microscope (microscope: Olympus SX7; light: Olympus DF PLAPO 1X_−4_, Olympus Optical, Tokyo, Japan) and photographed using a digital colour camera (Olympus SC 100, Olympus, Tokyo, Japan).

Seeds were categorised by size (seed length) as follows [9]: very small (100–200 µm), small (200–500 µm), medium (500–900 µm), large (900–2000 µm), and very large (2000–6000 µm). Seed pods were classified according to size as follows: very small (10–20 mm), small (20–30 mm), medium (30–40 mm), large (40–50 mm), and very large (>50 mm).

### 4.3. Assessment on Seed Micro-Morphometric

Seeds treated with tetrazolium chloride were measured under the microscope to determine seed size (length and width) and embryo size (length and width). Seeds with a length/width (SL/SW) above 5 were classified as elongated and those with an SL/SW below 5, as truncated. For each species, 50 seeds were measured, and the approximate seed, embryo and air-space volume were calculated following Prasongsom et al. [2], as below:

Embryo volume *:

(1) $$EV=\;\left(\frac{4}{3}\right)\times \left(\frac{22}{7}\right)\times \left(\frac{EL}{2}\right)\times {\left(\frac{EW}{2}\right)}^{2}$$

Seed volume:

(2) $$SV=2\times \left({\left(\frac{SW}{2}\right)}^{2}\times \left(\;\frac{SL\;}{2}\right)\times \left(1.047\right)\right)$$

Air-space volume:

(3) $$ASV=\frac{\left(\mathrm{SV}\;-\;\mathrm{EV}\;\times 100\right)}{SV}$$

SW = Seed width, SL = Seed length, EW = Embryo width, EL = Embryo length.

* For the measurement of embryo volume, we used the equation of Prasongsom et al. [2] for prolate-spheroid-shaped embryos, which all seeds in this study had [6]. The equation may change for oblate-spheroid embryos.

### 4.4. Statistical Analysis

**Figure 2:**
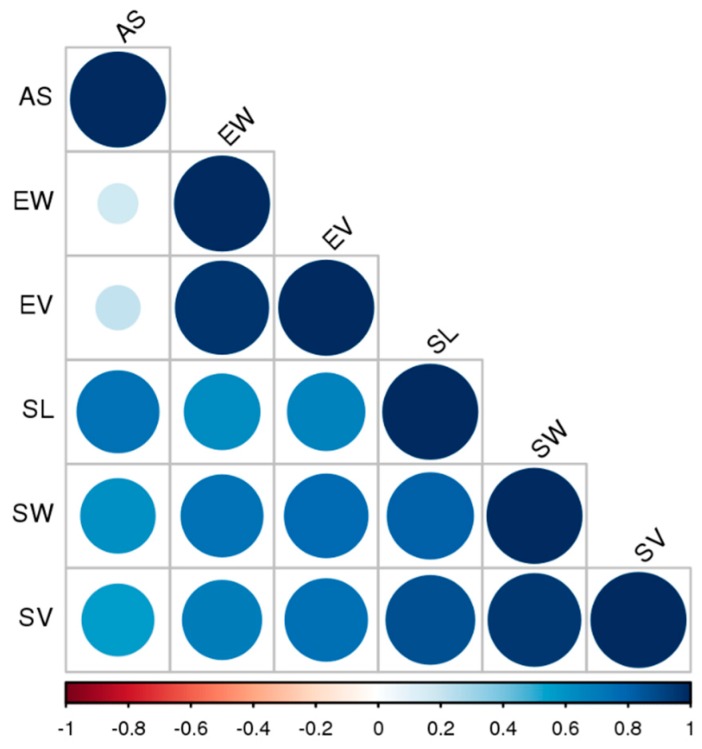
Correlation matrix between the studied morphological aspects. AS = air-space, EW = embryo width, EV = embryo volume, SL = seed length, SW = seed width, and SV = seed volume.

To evaluate micromorphological differences between the seeds, we used a Multivariate General Linear Model (GLM) using the taxonomy (subfamily), distribution range (temperate or tropical), habit (epiphytic or terrestrial) and species as independent variables. To fulfil the assumptions of the model, univariate and multivariate outliers were removed, and data were transformed using a natural logarithm (Ln) to meet (or approximate) normality. Since the model requires a moderate linear relationship, a correlation matrix was generated (Figure 2), and two variables, seed volume (SV) and embryo volume (EV), were removed from the model since they had a strong positive correlation (R^2^ > 90) with seed weight (SW) and embryo weight (EW), respectively. Pairwise post-hoc tests (LSD) were used to identify differences between species for each individual trait. Calculations were performed using IBM SPSS Statistics for Macintosh, Version 24.0. (IBM Corporation, Armonk, NY).

**Figure 3:**
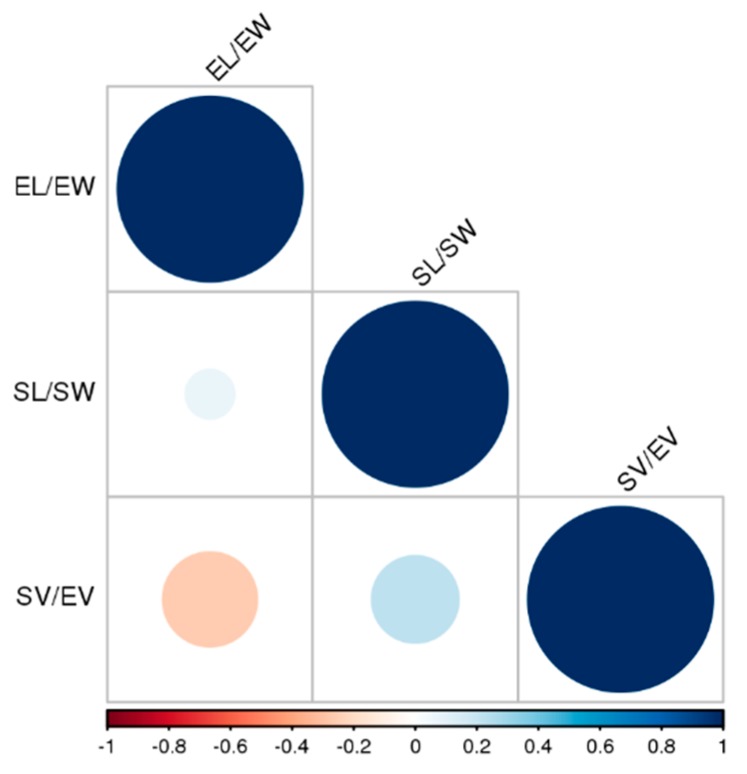
Correlation matrix between measurement ratios: EL = Embryo length, EW = embryo width, SL = seed length, SW = seed width, SV = seed volume, and EV = embryo volume.

We also calculated the relationship (ratio) between the different parameters investigated, i.e., EL/EW, SL/SW, and SV/EV. Then, we analysed them in the same way as described above. We found little correlation among these variables (Figure 3), so they were all included in the final model.

## 5. Conclusions

Our results show some similarities in qualitative traits (pod colour and size and seed colour) among taxonomically related orchid species. However, there was high variation in orchid seed morphometrics, likely because of ecological adaptations to their unique habitats and modes of dispersal. Information on orchid seed morphometrics is useful to infer the optimal dispersal mode, related to *in situ* conservation strategies and may help identify vulnerable species and set priorities for *ex situ* conservation. Further research including additional orchid species is needed to validate these results, and to explore other properties of the studied seeds (e.g., lipid composition) to develop optimal storage protocols for seed banking.

## Figures and Tables

**Table 1:** Variation in colour and size of seed pods and seeds in two tropical and four temperate orchid species.

Species	Seed Pod Length (cm)	Mature Pod Colour	Seed Colour
Tropical epiphytic			
*Dendrobium strebloceras*	6–7	Yellowish green	Whitish yellow
*Dendrobium lineale*	3.5–4	Yellowish green	Yellowish golden
Temperate epiphytic			
*Dendrobium cunninghamii*	1–1.5	Yellowish green	Brownish yellow
Temperate terrestrial			
*Gastrodia cunninghamii*	2–2.5	(Dark) brown	Brownish (light brown)
*Pterostylis banksii*	2–2.5	(Whitish) brown	Ochre (deep orange-brown)
*Thelymitra nevosa*	1.5–2	(Purplish) brown	Dark brown

**Table 2:** A comparative measurement of seed micromorphological characteristics of six selected orchid species and post-hoc comparisons after a GLM.

Embryo Traits				
Species	Length (mm)	Width (mm)	Volume *(mm^3^)	
*D. strebloceras*	0.25 ± 0.007 a	0.16 ± 0.005 a	0.004 ± 0.0003 a	
*D. lineale*	0.20 ± 0.004 d	0.097 ± 0.001 d	0.001 ± 0.00003 d	
*D. cunninghamii*	0.21 ± 0.004 c	0.099 ± 0.002 d	0.001 ± 0.00004 d	
*G. cunninghamii*	0.23 ± 0.005 b	0.097 ± 0.001 d	0.001 ± 0.00004 d	
*P. banksii*	0.21 ± 0.004 c	0.13 ± 0.002 c	0.002 ± 0.0001 c	
*T. nervosa*	0.24 ± 0.003 a, b	0.15 ± 0.002 b	0.003 ± 0.0001 b	
**Seed Traits**				
	**Length (mm)**	**Width (mm)**	Volume *(mm^3^)	**Air-space (%)**
*D. strebloceras*	1.76 ± 0.04 a	0.32 ± 0.009 a	0.052 ± 0.003 a	92.4 ± 0.4 a
*D. lineale*	0.42 ± 0.006 f	0.12 ± 0.002 d	0.001 ± 0.00006 d	32.2 ± 2 d
*D. cunninghamii*	0.48 ± 0.008 e	0.16 ± 0.003 b	0.003 ± 0.0002 c	66.2 ± 1.7 c
*G. cunninghamii*	0.85 ± 0.02 c	0.13 ± 0.002 c	0.004 ± 0.002 c	66.7 ± 1.2 c
*P. banksii*	1.09 ± 0.03 b	0.17 ± 0.003 b	0.009 ± 0.0004 b	75.3 ± 1.2 b
*T. nervosa*	0.54 ± 0.006 d	0.17 ± 0.003 b	0.004 ± 0.0001 c	29.3 ± 1.5 d

* We ran a separate ANOVA followed by LSD for embryo and seed volume (Log transformed), since these variables were excluded from the original model. Different letters indicate significant differences (*p* < 0.05) between species for each individual trait (mean ± SE) after a pairwise (LSD) post-hoc test.

**Table 3:** Calculated ratios for embryo length to embryo width (EL/EW), seed length to seed width (SL/SW), and seed volume to embryo volume (SV/EV) for six orchid species and post-hoc comparisons after a GLM.

Species	EL/EW	SL/SW	SV/EV
*D. strebloceras*	1.55 ± 0.3 d	5.6 ± 0.1 c	15.7 ± 1.2 a
*D. lineale*	2.02 ± 0.4 c	3.7 ± 0.8 d	1.58 ± 0.08 c
*D. cunninghamii*	2.13 ± 0.4 b	2.9 ± 0.7 f	3.4 ± 0.2 b
*G. cunninghamii*	2.36 ± 0.5 a	6.7 ± 0.2 a	3.2 ± 0.1 b
*P. banksii*	1.61 ± 0.3 d	6.4 ± 0.2 b	4.9 ± 0.5 b
*T. nervosa*	1.64 ± 0.3 d	3.29 ± 0.5 e	1.4 ±0.03 c

Different letters indicate significant differences (*p* < 0.05) between species for each trait (mean± SE) after a pairwise (LSD) post-hoc test.

## Supplementary Materials

The following are available online at https://www.mdpi.com/2223-7747/9/2/161/s1, Table S1: The plant taxonomy, morphology, biogeographical origin, and ecological habits for each species.
