# Supplementary material for: Comparative Seed Morphology of Tropical and Temperate Orchid Species with Different Growth Habits
Source: Plants (Basel). 2020 Jan 29;9(2):161. doi: 10.3390/plants9020161 (PMC7076704; doi:10.3390/plants9020161)
Supplement: Supplementary file 1 [file plants-09-00161-s001.pdf]

Table S1. Plant taxonomy, morphology, biogeographical origin and ecological habits of the orchid species used in this study.

Species (Common Name)	Sub Family/ Tribe/Genus/ Section	Distribution	Habitat & Morphology	Flowering Time/Flower Duration/ Capsule Maturity Time	Flower	Reference(s)
Dendrobium strebloceras (Twisted horn *Dendrobium*)	Epidendroideae/ Dendrobiinae/ *Dendrobium*/ *Spatulata* Lindl.	Indonesia. The Moluccas, Halmahera Island	Habitat: tropical epiphytic, rain forests, low altitudes, warm conditions, high humidity, bright-filtered light<br>Morphology:<br>- Large-sized plant<br>- Pseudobulbs: up to 1.5 m long, 1.5 cm in diameter<br>- Leaves: fleshy, ovate-oblong to lanceolate along the upper half, up to 16 cm long, 5 cm wide<br>- Inflorescence: up to 40 cm long, 6–8 flowers, each up to 5 cm long, fragrant<br>- Petal and sepal: twisted, pale yellow or green suffused with brown to dark brown, darkens with age	Throughout the year/up to 2 months/150 days	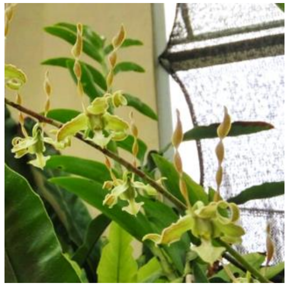<br>(Photo: Suskandari)	[1]
Dendrobium lineale (Morobe shower orchid)	Epidendroideae/ Dendrobiinae/*D endrobium*/ *Spatulata* Lindl.	Indonesia. Irian Jaya border to Milne Bay (North-eastern coast of New Guinea)	Habitat: Tropical epiphytic/lithophytic, rain forests, low altitudes (coastal or water streams at sea level to above 800 m), warm conditions, high humidity, direct sunlight<br>Morphology:<br>- Large-sized plant<br>- Pseudobulbs: up to 2 m long and 2-3 cm in diameter<br>- Leaves: fleshy, leathery oblong to lanceolate along the upper two-thirds of stem, 8–15 cm long<br>- Inflorescence: up to 75 cm long, arching, flowers up to 5 cm across<br>- Petal and sepal: white, pale yellow–purplish vein predominating lip, dark brown at edge of lower petals	Throughout the year/ up to 2–3 months/ 120 days	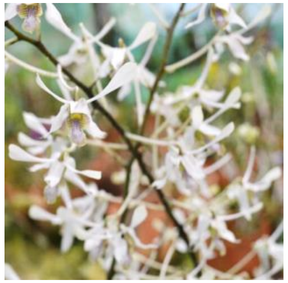<br>(Photo: Suskandari)	[1]

Dendrobium cunninghamii (Christmas orchid, bamboo orchid, winika, pekapeka)	Epidendroideae/ Dendrobiinae/ *Winika*/ -	New Zealand (endemic). North and South Islands, the Chatham & Stewart Island	Habitat: Temperate epiphytic/lithophytic, moist rain forests, low altitudes below 500 m), intermediate-cool temperature, semi-shade<br>Morphology:<br>- Pseudobulbs: long, slender, yellowish canes up to 2 m long, branched, pendulous, clustering in large clump<br>- Leaves: leaf sheath throughout stem, lateral, borne on the upper half of final branches of the stem, 2–3 cm long, 3–3.5 mm wide<br>- Inflorescence: lateral, 3-6 flowers, each up to 2.5 cm across<br>- Petal and sepal: white-yellow base with purple column wings, 4 yellow-green purple topped ribs in the mid-lamina, membranous segments, 3-lobed lip, broad labellum	December-January/ up to 10 days/ 1 month	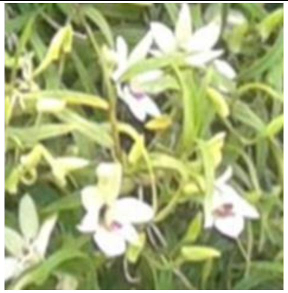<br>(Photo: Paul Topp)	[1,2]
Gastrodia cunninghamii (Potato orchid)	Epidendroideae/ Gastrodieae/ *Gastrodia*/ -	New Zealand. East coast of North and South Island, Middle and lower part of South Island, the Chatham & Stewart Island	Habitat: temperate, terrestrial, saprophytic, lowland to montane forests, common in mature pine plantations<br>Morphology:<br>- Tall plant (up to 1 m height)<br>- Tuber: Large and extensively branched<br>- Leaves: no leaves, no chlorophyll, plant is brown, black or greenish with tiny scales on the stem as residual leaves<br>- Inflorescence: up to 70 flowers per stem, knobbly and tubular flowers<br>- Petal and sepal: petals and sepals are merged and separated only at their tips, labellum is attached at the inner base of the tube, only black tipped yellow end shows	November-February/ up to 2 months/ information not available	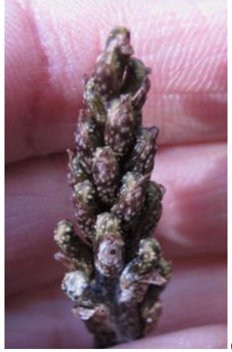 (Photo: Surya Diantina)	[1,2,3]
Pterostylis banksii (Greenhood orchid)	Orchidoideae/ Pterostylidinae/ *Pterostylis*/ -	New Zealand. North and South Islands (except some part of east-coast and middle of South Island), the Chatham & Stewart Island	Habitat: Temperate, terrestrial, lowland to montane and subalpine forests, scrub<br>Morphology:<br>- Tuber: ovoid, form dense colonies, plant height up to 50 cm (one of New Zealand's largest orchids)<br>- Leaves: thin grass-like leaves, usually 4-6, up to 25 cm in length, long and smooth, internodes very short near the base, distributed up the stem<br>- Petal and sepal: single flower, 3-5 cm high, green with translucent white stripes, tips of petals range in colour from white to orange-red, dorsal sepal very long, often slightly upturned at the tip. Petals much shorter than the dorsal sepal. Lateral sepals diverge at a narrow angle, tips very long, erect or swept backward. Labellum tip is flat or arched, top reddish. Stigma long and narrow.<br>(Notes: Size, structure, and colouring can be quite variable)	October - December/ 1 month (*P. patens*)/ 2 months (*P. patens*)	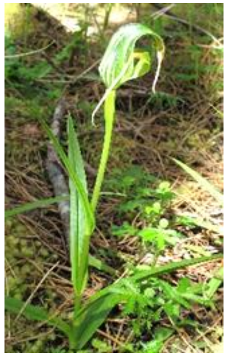<br>(Photo: Surya Diantina)	[1,2,3,4]

Thelymitra nervosa (Sun orchid)	Orchidoideae/ Diurideae/ *Thelymitra*/ -	New Zealand. Several parts of central and lower North Island, upper part and few areas on the east coast of South Island, the Chatham Island	Habitat: Temperate, terrestrial, open areas, low vegetation often on clay, wet, peaty areas<br>Morphology:<br>- Tuber: ovoid (oval)<br>- Leaf: single, long, narrow and thin leaf, quite fleshy, channelled and keeled, sheathering the stem at its base<br>- Inflorescence: bright colour flower, blooms in warm sunny days, regular shape, short column, grows up to 30 cm, up to 6 flowers of 1.5 cm in diameter<br>- Petal and sepal: dark lavender-blue or pink, unstriped, petal spotted, column blue with thick erect white hairs on the horizontal arms, dark brown slightly hooded midlobe, warty with dark tubercles and yellow toothed rim	October - January/ 5 days (*T. longifolia*)/ information not available	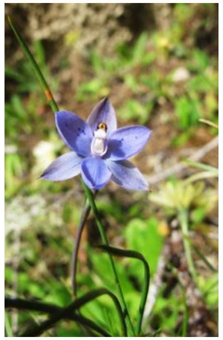<br>(Photo: Surya Diantina)	[1,2,3,4]

References

1. Lavarack, P.S.; Harris, W.F.; Stocker, G. *Dendrobium and its relatives*. Timber Press: US; 2000.
2. New Zealand Native Orchid Group. *A pocket guide to the New Zealand native orchids*. The New Zealand Native Orchid Group Inc.: wellington, New Zealand; 2015.
3. George, st I. The natural guide to New Zealand orchids. Random House: Auckland, New Zealand, 1999; 176 p.
4. Lehnebach, C.; Robertson, A.W.; Hedderley, D. Pollination of four NZ terrestrial orchids. *N. Z. J. Bot.* **2005**, *43*, 467–477. doi:0028–825X/05/4302–0467.
